# Elevated H2AX Phosphorylation Observed with kINPen Plasma Treatment Is Not Caused by ROS-Mediated DNA Damage but Is the Consequence of Apoptosis

**DOI:** 10.1155/2019/8535163

**Published:** 2019-09-19

**Authors:** Sander Bekeschus, Clarissa S. Schütz, Felix Nießner, Kristian Wende, Klaus-Dieter Weltmann, Nadine Gelbrich, Thomas von Woedtke, Anke Schmidt, Matthias B. Stope

**Affiliations:** ^1^ZIK plasmatis, Leibniz Institute for Plasma Science and Technology (INP Greifswald), Greifswald, Germany; ^2^Department of Urology, Greifswald University Medical Center, Greifswald, Germany; ^3^Institute for Hygiene and Environmental Medicine, Greifswald University Medical Center, Greifswald, Germany

## Abstract

Phosphorylated histone 2AX (*γ*H2AX) is a long-standing marker for DNA double-strand breaks (DSBs) from ionizing radiation in the field of radiobiology. This led to the perception of *γ*H2AX being a general marker of direct DNA damage with the treatment of other agents such as low-dose exogenous ROS that unlikely act on cellular DNA directly. Cold physical plasma confers biomedical effects majorly via release of reactive oxygen and nitrogen species (ROS). *In vitro*, increase of *γ*H2AX has often been observed with plasma treatment, leading to the conclusion that DNA damage is a direct consequence of plasma exposure. However, increase in *γ*H2AX also occurs during apoptosis, which is often observed with plasma treatment as well. Moreover, it must be questioned if plasma-derived ROS can reach into the nucleus and still be reactive enough to damage DNA directly. We investigated *γ*H2AX induction in a lymphocyte cell line upon ROS exposure (plasma, hydrogen peroxide, or hypochlorous acid) or UV-B light. Cytotoxicity and *γ*H2AX induction was abrogated by the use of antioxidants with all types of ROS treatment but not UV radiation. H2AX phosphorylation levels were overall independent of analyzing either all nucleated cells or segmenting *γ*H2AX phosphorylation for each cell cycle phase. SB202190 (p38-MAPK inhibitor) and Z-VAD-FMK (pan-caspase inhibitor) significantly inhibited *γ*H2AX induction upon ROS but not UV treatment. Finally, and despite *γ*H2AX induction, UV but not plasma treatment led to significantly increased micronucleus formation, which is a functional read-out of genotoxic DNA DSBs. We conclude that plasma-mediated and low-ROS *γ*H2AX induction depends on caspase activation and hence is not the cause but consequence of apoptosis induction. Moreover, we could not identify lasting mutagenic effects with plasma treatment despite phosphorylation of H2AX.

## 1. Introduction

*γ*H2AX is a recognized marker for DNA double-strand breaks (DSBs) in radiation biology [1]. Phosphorylation at serine139 of the histone 2AX occurs rapidly, and approximately 1% of all H2AX proteins are phosphorylated per gray irradiation via a molecular machinery [2]. Based on these findings in radiobiology, *γ*H2AX has been used as direct surrogate and correlate of DNA DSBs in a variety of studies testing chemical and physical treatments, for example, in the field of oncology [3]. One novel physical treatment modality for the treatment of cancer is cold physical plasma [4]. Its antitumor effects on several types of tumor cells such as skin cancer have been shown *in vitro* and *in vivo* [5–7], and to a limited extent also in small patient cohorts [8]. Medical plasmas are multicomponent systems consisting of, e.g., electrons and ions, electric fields, and a multiplicity of different reactive oxygen and nitrogen species (ROS) [9].

ROS are the major component mediating biomedical effects of plasma treatment, at least *in vitro* [10–12]. Concomitant with plasma-induced cell death, many studies reported a phosphorylation of H2AX with different kinds of plasma sources and (tumor) cell types [13–19]. This lead to the conclusion that plasma-derived ROS directly induce DNA damage. Yet, there are several pitfalls of this assumption. Firstly, plasma-derived ROS are not generated within the cells but reach them from the outside. Due to the charge as well as short lifetime of some of these species detected and quantified in plasma-treated liquids, and hence possibly in the vicinity of cells [20–22], only a fraction of the ROS is able to diffuse or being transported through the cell membrane directly. Secondly, once in the cytosol, there are abundant reaction partners including, for instance, peroxiredoxins (PRDX) to scavenge the ROS [23]. Thirdly, the remaining ROS would have to cross several membranes of the endoplasmic reticulum (ER) and ultimately the nucleus membrane to directly act on cellular DNA after—again—passing by several antioxidant proteins such as PRDX2 [24]. In the light of large distances (2-10 *μ*m) that ROS would have to travel from the membrane to the nucleus, the direct action of plasma-derived ROS on cellular DNA denies the nature of ROS being reactive and short-lived. This also questions the conclusion that *γ*H2AX is an indicator of DNA DSBs arising from primary ROS derived from plasma treatment. Rather, a role of *γ*H2AX might be in marking DNA DSBs secondary to plasma treatment (e.g., due to apoptosis). *γ*H2AX was originally identified as an early event after the direct formation of DSBs. Now, *γ*-H2AX is considered to occur after the indirect formation of DSBs caused by cellular process such as DNA repair, replication, and/or transcription at sites of initial DNA damage such as oxidative bases, DNA adducts, single-strand breaks, cross-linking, and DNA photoproducts [25, 26].

In general, *γ*H2AX seems to fulfill pleiotropic roles in cell biology. For instance, low levels of *γ*H2AX are not associated with DNA DSBs [27]. Untreated cells are found to be positive for *γ*H2AX in the M phase of the cell cycle without being exposed to a DNA-damaging agent [28]. Decreased expression of the H2AX primarily leads to damage in the mitochondria [29]. The amount of *γ*H2AX in apoptotic cells also exceeds that of nonapoptotic cells by a factor of ten [30]. *γ*H2AX also seems dispensable for the initial recognition of DNA breaks [31]. Finally, there is evidence that not only DNA DSBs but also ROS may be responsible for *γ*H2AX induction [32]. Mechanistically, it is known that while the serine/threonine kinase ataxia telangiectasia mutated (ATM) forms *γ*H2AX at DNA DSBs [33], the serine/threonine kinase ataxia telangiectasia and Rad3-related protein (ATR) have been also implicated in this process [34]. Interestingly, ATM does so by redox-sensitive thiols, so ATM activation is a marker of both oxidative stress and DNA DSBs [35].

In the light of these studies, we sought to study the role of plasma-derived ROS in *γ*H2AX induction. The lymphocyte cell line TK6 was treated with an atmospheric pressure argon plasma jet (kINPen) as ROS-source. The *γ*H2AX expression along with the amount of ROS, ROS scavengers, cell viability, and an OECD- (Organization for Economic Co-operation and Development-) accredited genotoxicity (micronucleus) assay were investigated. We found that plasma but not UV-induced *γ*H2AX induction was dependent on apoptosis and caspase activation, making DNA damage marked via *γ*H2AX rather a consequence than the cause for plasma-induced cell death.

## 2. Materials and Methods

### 2.1. Cell Culture

Lymphocytes are the cell type most often used when investigating DNA damage [36]. Especially the human TK6 lymphocyte cell line has been widely utilized in genotoxicity studies [37, 38]. For this reason, we used TK6 (ATCC CRL-8015) cells, a p53-competent, human lymphoblast cell line. Cells were cultured in Roswell Park Memorial Medium without phenol red (RPMI1640; PanBioTech) supplemented with 10% fetal bovine serum, 2% glutamine, and 1% penicillin/streptomycin (all Sigma). All incubations were performed in cell culture conditions (*CB210*; Binder) at 37°C, 95% humidity, and 5% carbon dioxide. As ROS scavengers, catalase (cat; 20 *μ*g/ml), glutathione (GSH; 1 mM), or superoxide dismutase (SOD; 100 U/ml) was used (all Sigma). As enzyme or signaling inhibitors, Z-VAD-FMK (R&D Biosciences), SB202190 (Sigma), KU55933 (SelleckChem), Ly294002 (Cell Signaling Technologies), wortmannin (InvivoGen), or SP600125 (Santa Cruz Biotechnology) was used at different concentrations and incubated with cells for 1 h prior ROS or UV treatment. Final concentrations for a selected choice of inhibitors were 1 *μ*M for KU55933, 1 *μ*M for SB202190, and 25 *μ*M for Z-VAD-FMK.

### 2.2. Exposure of Cells to ROS, Cold Physical Plasma, or UV

**Figure 1:**
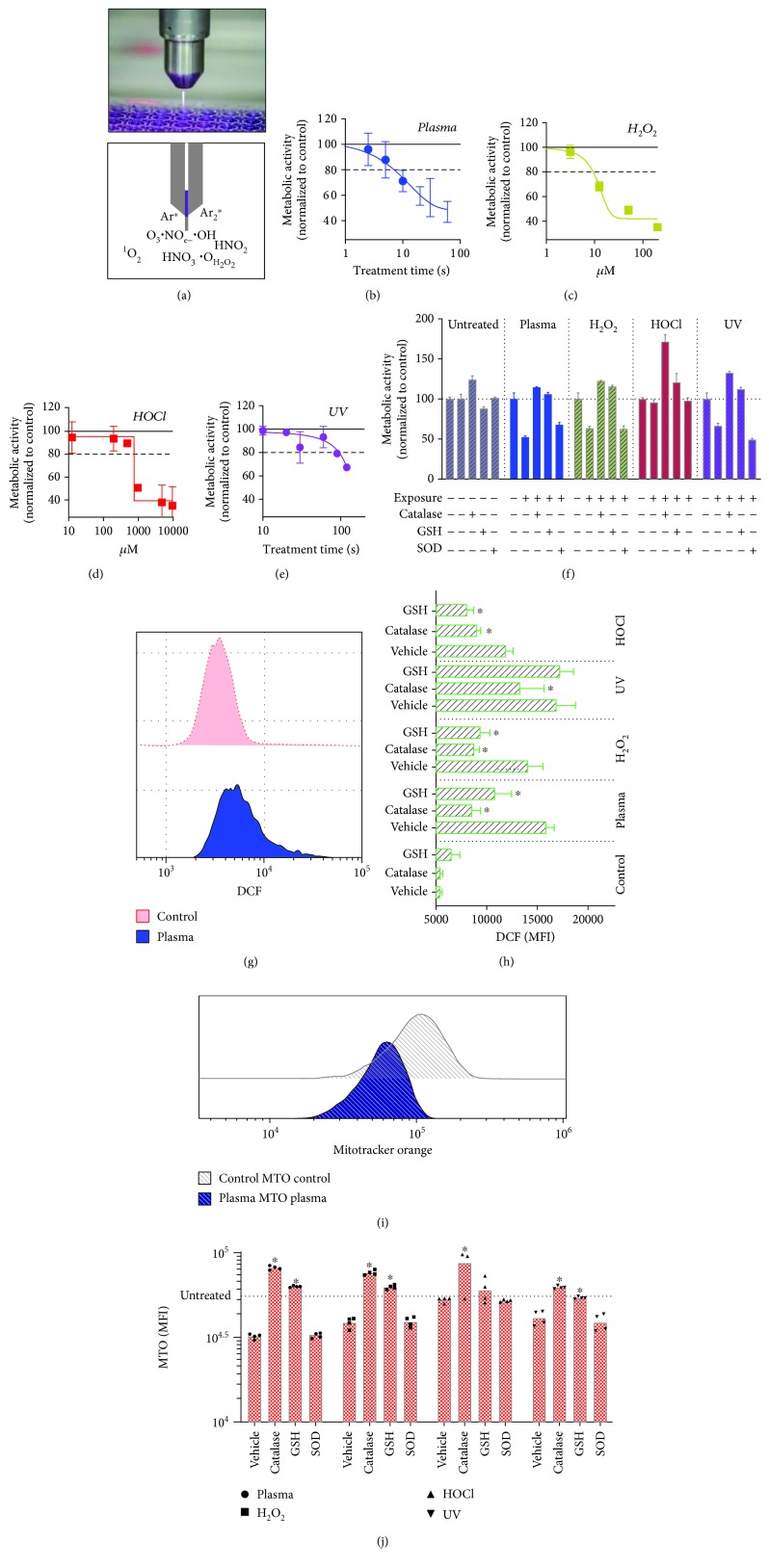
Metabolic activity and oxidation of TK6 cells after exposure to plasma, H_2_O_2_, HOCl, and UV. (a) Image (top) and scheme (bottom) with some of the products generated by the kINPen argon plasma jet. (b–e) Metabolic activity 6 h after exposure to different concentrations of ROS, or plasma or UV treatment times. (f) Effects of antioxidants or ROS scavenging enzymes on the metabolic activity of cells in responses to treatments after 6 h. (g) Overlay histogram of DCF fluorescence of control and plasma-treated cells. (h) Quantification of DCF fluorescence in cells immediately after treatment in the presence or absence of antioxidants. (i) Overlay histogram of mitotracker orange (MTO) in cells 6 h after plasma treatment. (j) Quantification of mitochondrial mass in cells exposed to various agents in the presence or absence of antioxidants. Data are mean + S.E. of 2–4 independent experiments with several replicates each. Statistical analysis (h, j) within each treatment group was done with one-way ANOVA and Dunnett's post hoc test to vehicle control.

For all procedures, 2.5 × 10^5^ TK6 cells in 500 *μ*l of fully supplemented cell culture medium were added to wells of a 24-well plate (Sarstedt). For hydrogen peroxide (H_2_O_2_; Sigma) treatment, the stock was diluted in double-distilled water, and a range of concentrations was tested initially. Final concentration for subsequent assays was 10 *μ*M. For hypochlorous acid (HOCl; Roth), the stock was diluted in double-distilled water, and a range of concentrations was tested initially. Final concentration for subsequent assays was 500 *μ*M. Plasma treatment was done using the atmospheric pressure argon plasma jet kINPen (neoplas tools) that expels various reactive agents (Figure 1(a)) as reported before [9]. Its biomedical effects were summarized recently [39]. The plasma source was operated at two standard liters per minute of argon gas (Air Liquide, purity 99.999%). Plasma treatment was performed in a highly standardized manner as shown previously [40]. Briefly, the plasma jet was attached to a computer-controlled *xyz*-table (CNC step), which hovered the plasma exactly over the center of each well for a predefined time and height. A range of treatment times was initially tested. Immediately after treatment, a predetermined amount of double-distilled water was added to the wells after plasma treatment to compensate for evaporation effects and to maintain isoosmolality. A treatment time of 10 s was used for subsequent experiments if not indicated otherwise. For exposure to UV light, a range of exposure times was tested, and 120 s was used for experiments if not indicated otherwise. The wells of the plate that were not intended to be exposed to UV light were covered with aluminum foil. A broadband UVB (20-160 J_eff_ m^−2^) light source (Philips TL12 fluorescent lamp) emitting radiation between 290 and 315 nm was used. UVB exposure modifies DNA directly by forming cyclobutane pyrimidine dimers (CPD) and 6-4 photoproducts. Indirect effects of UVB on DNA occur due to photolysis and generation of hydroxyl radicals, leading to formation of 8-hydroxy-2′-deoxyguanosine (8-OhdG) [41].

### 2.3. Intracellular Oxidation

To assess intracellular oxidation, TK6 cells were stained with chloromethyl 2′,7′-dichlorodihydrofluorescein diacetate (CM-H_2_DCF-DA; final concentration 2.5 *μ*M; Thermo Fisher) in phosphate-buffered saline (PBS), washed, and resuspended in fully supplemented cell culture medium (in the presence or absence of antioxidants). Cells were seeded into plates and treated as described above. Immediately after, cells were added to 12 × 75 mm tubes (Sarstedt) containing 4′,6-diamidin-2-phenylindol (DAPI; final concentration 1 *μ*M; Sigma), and samples were acquired by multicolor flow cytometry (*Gallios*, equipped with 405 nm, 488 nm, and 638 nm laser; Beckman Coulter). Sample analysis was performed using Kaluza 2.1.1 software (Beckman Coulter) and analyzing the mean fluorescent intensity (MFI) of DCF within the viable (DAPI-) cell fraction.

### 2.4. Metabolic Activity, Mitochondrial Mass, and Viability

Metabolic activity was investigated by incubating the cells for 4 h with resazurin (final concentration 100 *μ*M; Alfa Aesar) after two hours of incubation posttreatment. Metabolically active cells transform nonfluorescent resazurin into fluorescent resorufin, which can be quantified using a multiplate reader (*F200*; Tecan) at *λ*_ex_ 560 nm and *λ*_em_ 590 nm. Absolute sample values were normalized to that of untreated cells = 100%. To quantify mitochondrial mass, cells were incubated for 15 min with chloromethyltetramethylrosamine, also called MitoTracker Orange (MTO; final concentration 1 *μ*M; Thermo Fisher), at 6 h after plasma treatment. The cationic rosamine probe only binds to mitochondrial membranes with intact potential. Sample acquisition was performed using flow cytometry. To quantify nonterminally dead (alive) and terminally dead cells, DAPI was used to discriminate the percentage of either population using flow cytometry. For some experiments, the amount of cells active for caspase 3 and 7 was investigated to quantify the amount of apoptotic cells. For this, cells were incubated for 30 min with CellEvent dye (final concentration 2.5 *μ*M; Thermo Fisher). Samples were analyzed by flow cytometry.

### 2.5. Analysis of Cell Cycle and *γ*H2AX

Flow cytometry is the most sensitive, quantitative, and informative method of analyzing and quantifying *γ*H2AX in cells, as it can be related to cell cycle and other cellular populations stained with additional markers [36]. To prepare the cells for DNA and *γ*H2AX staining, cells were harvested 2 h after exposure to agents into 12 × 75 mm tubes. In an initial kinetic experiment, 2 h was shown to be optimal. Cells were washed with PBS and fixed with -20°C methanol for 30 min at 4°C. Cells were washed and incubated with murine phosphor-specific anti-*γ*H2AX antibodies (BioLegend) for 20 min. The optimal antibody dilution was determined experimentally. Cells were washed and incubated with DAPI (10 *μ*M) and an anti-mouse IgG1 antibody conjugated to Alexa Fluor 647 (Thermo Fisher) for 20 min in permeabilization wash buffer (BioLegend) in the dark. Cells were washed and resuspended in PBS and acquired by flow cytometry. Gating of cells was performed as shown. Appropriate gating of the DAPI-area vs. DAPI-width parameters for cell cycle analysis was confirmed with Michael H. Fox algorithm integrated into Kaluza analysis software. If the algorithm could not calculate G1, S, and G2 phase properly, the gating was adjusted accordingly. This way, *γ*H2AX induction could be accurately calculated in relation to mathematical modeling for each cell cycle phase. A total of more than 1.800 single FACS measurements was prepared, stained, and individually acquired in this study. Each measurement contained at least 20,000 single cells, yielding quantitative single cell data. To analyze *γ*H2AX foci via laser scanning confocal microscopy (TP5; Leica), cells were stained as described above and added to 8-well glass slides (Ibidi). Fluorescence was acquired using excitation at 405 nm for DAPI and 640 nm for Alexa Fluor 647.

### 2.6. Quantification of Micronuclei

The cytokinesis-block micronucleus (MN) assay requires quantification of micronuclei in binucleated cells (BNCs) only [42]. Cells were treated as described above with minor changes and incubated for 24 h. As additional genotoxic positive control, the DNA-damaging agent methyl methanesulfonate (MMS; final concentration 20 ng/ml; Sigma) was added. The plasma treatment time was reduced from 10 s to 2.5 s as the plasma treatment acted synergistically toxic together with cytochalasin B, leading to insufficient cell counts. Similar observations were made for UV treatment, which was reduced from 120 s to 24 s. Ten wells were pooled into T75 flasks (Sarstedt) per condition, and cytochalasin B (final concentration 5 *μ*g/ml; Sigma) was added. Flasks were incubated for another 24 h. Cells were collected into 15 ml tubes (Sarstedt), washed, fixed with 4% fixation buffer (BioLegend) for 20 min, and washed and stored at 4°C in PBS until staining. For staining, cells were washed and stained in permeabilization wash buffer (BioLegend) containing draq5 (final concentration 50 *μ*M; BioLegend) for 20 min at room temperature in the dark. Other DNA staining dyes were also compared (SYTOX green, final concentration 1 *μ*M, Thermo Fisher; DAPI, final concentration 10 *μ*M, Sigma; Hoechst 33342, final concentration 10 *μ*g/ml, Sigma) but found to be less suitable. Cells were washed in permeabilization wash buffer and resuspended in PBS in siliconized 1.5 ml tubes. Speed beats (Merck Millipore) were used to operate the imaging fluids of an ImageStream ISX Mark II (Merck Millipore), which was used for sample acquisition. Up to 2 × 10^5^ cells (images) were acquired per sample. The digital MN analysis was in main parts similar as reported before [43], with some minor modifications in mask design and gating steps. A total of more than 40 Mio single cells—each represented by at least two individual images of about 200 × 200 pixels in size—were acquired and partly analyzed in this study.

### 2.7. Statistical Analysis

Data were analyzed and graphed using Prism 8.1 (GraphPad software). Mean and standard error (S.E.) were given if not indicated otherwise. Statistical analysis was performed either using one-way analysis of variances or *t*-test.

## 3. Results

### 3.1. Viability and Oxidation upon Exposure to Plasma, H_2_O_2_, HOCl, and UV Light

In order to obtain ROS concentrations and UV exposure times as well as plasma treatment times that were neither too toxic nor failed to show effects on cells, dilution and treatment time series were performed, respectively. From these, we concluded to use 10 s of plasma treatment (Figure 1(b)), 10 *μ*M of H_2_O_2_ (Figure 1(c)), 500 *μ*M of HOCl (Figure 1(d)), and 120 s of UV treatment (Figure 1(e)) in viability experiments assayed 6 h after exposure for subsequent experiments. Data for metabolic activity were in principal similar for longer incubation times (Supplementary [Supplementary-material supplementary-material-1]). Terminally dead cells at 6 h posttreatment were also quantified (Supplementary [Supplementary-material supplementary-material-1]). Next, it was tested whether antioxidant agents and enzymes protected cells 6 h postagent-induced toxicity (Figure 1(f)). While catalase (cat) and glutathione (GSH) conferred protection, superoxide dismutase (SOD) did not. To confirm that this finding was related to protection from ROS, cells were stained with CM-H_2_DCF-DA, which after intracellular modifications fluoresces upon oxidation with, e.g., plasma treatment (Figure 1(g)). Indeed, GSH and cat significantly protected cells from oxidation with plasma, H_2_O_2_, and HOCl treatment (Figure 1(h)). For UV exposure, only cat but not GSH conferred protection. This might be due to UV radiation directly oxidizing DCFH-DA. In lymphocytes, ROS-induced toxicity leads to depolarization of the mitochondrial membrane potential ΔΨm [44], which allows the quantification of mitochondria with intact ΔΨm using appropriate dyes (Figure 1(i)). The agents decreased the total amount of mitochondria with intact ΔΨm, while cat and GSH but not SOD protected from insult (Figure 1(j)). Presence of cat even led to higher values, suggesting the growth-supporting activity of antioxidant enzymes. In summary, the ROS agents and UV radiation oxidized the cells leading to mitochondrial damage and reduction of metabolic activity and viability.

### 3.2. Induction of *γ*H2AX Depended on ROS but Not of Cell Cycle Phase

**Figure 2:**
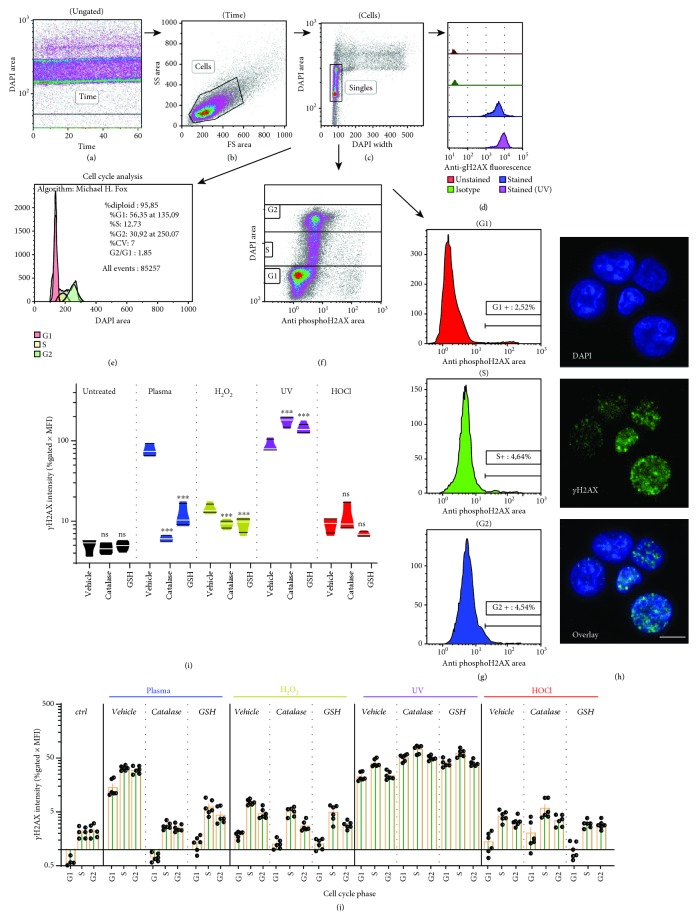
Analysis of *γ*H2AX in TK6 cells and its relation to ROS. (a–c) Gating strategy of TK6 cells at 2 h after treatment with agents was done by first including cells in time (a) and forward (FS) and side scatter (SS) cell gate (b), before excluding doublets and subG1 cells for the singles gate (c). (d–e) Singles were then analyzed for total *γ*H2AX as exemplified with representative fluorescence histogram overlay (d), subjected to algorithm-driven cell cycle analysis (e), or manually gated for each cell cycle phase (f) and subsequent determination of *γ*H2AX^hi^ cells in histograms (g). (h) Confirmation of *γ*H2AX foci (green) in DAPI-stained nuclei (blue) by confocal laser scanning microscopy. (i) Quantification of total (independent of cell cycle phase) *γ*H2AX with treatments and presence or absence of antioxidants. (j) Quantification of total *γ*H2AX within each cell cycle phase at 2 h after treatment with agents in the presence or absence of antioxidants. Quantification (i, j) was done by multiplying the percent of cells positive for *γ*H2AX (% gated) with the mean fluorescent intensity (MFI) of *γ*H2AX^+^ cells. Data show violin plots (i) or single values and mean ± S.E. (j) of three independent experiments with duplicates each. Statistical analysis (i) within each treatment group was done with one-way ANOVA and Dunnett post hoc test to vehicle control. Scale bar (h) is 10 *μ*m; ns = not significant.

To quantify *γ*H2AX in cells, a rigid flow cytometric gating strategy was set up. Cells were gated based on time (Figure 2(a)) and forward and side scatter properties (Figure 2(b)) followed by exclusion of doublets, aggregates, and subG1 cells (Figure 2(c)). *γ*H2AX was quantified in singlets (Figure 2(d)). Alternatively, *γ*H2AX was determined per cell cycle phase, which was validated using Michael H. Fox algorithms (Figure 2(e)). For each phase, a separate gating was applied (Figure 2(f)), from which the number (% gated) and intensity (mean fluorescence intensity of % gated) was calculated (Figure 2(g)). Staining was performed using appropriate antibody dilutions (Supplementary [Supplementary-material supplementary-material-1]) at 2 h posttreatment (Supplementary [Supplementary-material supplementary-material-1]) and was confirmed using confocal laser scanning microscopy (Figure 2(h)). *γ*H2AX foci are formed within seconds, but since they are initially quite small, reliably quantification is recommend earliest at 30 min after initial insult [45]. A prominent *γ*H2AX induction was observed with plasma and UV treatment and to a lesser extent with H_2_O_2_ and HOCl exposure (Figure 2(i)). This difference may be explained by slight (H_2_O_2_) and larger (HOCl) differences of the oxidants to induce cytotoxic effects as compared to those seen with plasma (Figures 1(f) and 1(j)). The reason might have been a change of TK6 sensitivity between the initial titration (Figures 1(b) and 1(c)) and subsequent experiment due to passage number. Strikingly, antioxidants (GSH and cat) significantly reduced *γ*H2AX induction for plasma and H_2_O_2_ treatment. For UV treatment, it was significantly enhanced. Similar observations were made when analyzing *γ*H2AX induction for each phase of the cell cycle (Figure 2(j)). In general, G1 cells gave lower signals compared to S and G2 phase cells, and the increase observed with antioxidants in UV conditions was evenly proportional for each cell cycle phase. Notably, cat and GSH had no effect on *γ*H2AX induction in resting (untreated) cells for each cell cycle phase (Supplementary [Supplementary-material supplementary-material-1]). By contrast, the antioxidant N-acetylcysteine (NAC) increased *γ*H2AX induction in untreated as well as treated cells (Supplementary [Supplementary-material supplementary-material-1]). With reference to cell cycle phase-dependent *γ*H2AX intensity (Figure 2(j)), another question was whether there was a relatively higher increase in proliferating (S and G2 phase) cells. These cells have intrinsically more DSBs and unwinded DNA, which could make them more prone to ROS-induced DNA damage. It was observed that the opposite was the case, as *γ*H2AX intensity in S over G1 and G2 over G1 was overall significantly lower compared to those of untreated control cells (Supplementary [Supplementary-material supplementary-material-1]). Altogether, *γ*H2AX showed a major increase in plasma-treated cells, which was almost fully abrogated in presence of cat or GSH during the treatment.

### 3.3. Intracellular Signaling and Apoptosis Govern Plasma-Induced *γ*H2AX

**Figure 3:**
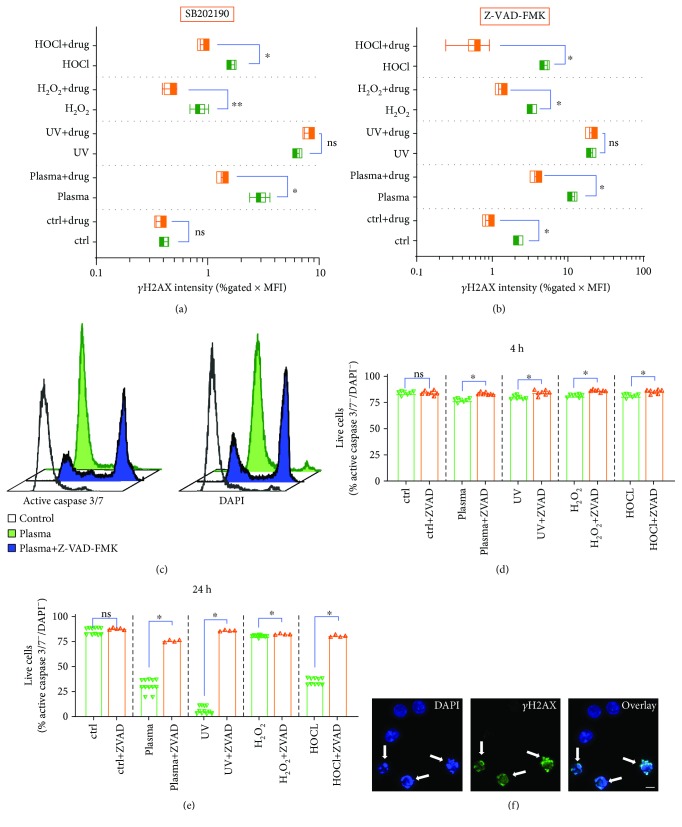
Dependence of ROS and UV-induced *γ*H2AX expression on intracellular signaling and apoptosis. (a, b) *γ*H2AX expression in cells preincubated with (a) SB202190 (p38 MAPK-inhibitor) or (b) Z-VAD-FMK (pan-caspase inhibitor) 2 h after exposure to various agents. (c) Representative overlay histograms of active caspase 3/7-stain (left) and terminally dead DAPI^+^ (right) in presence of absence of Z-VAD-FMK at 24 h after plasma treatment. (d–e) Quantification of apoptosis in presence or absence of Z-VAD-FMK at (d) 4 h and (e) 24 h after plasma treatment with *γ*H2AX-inducing agents. (f) Confocal laser scanning microscopy (DNA = DAPI, blue; *γ*H2AX = green) of TK6 cells with arrows pointing at apoptotic (cells with fragmented nuclei) cells being positive for *γ*H2AX. Data show box plots (a, b) and single data and mean (d, e) of two to four independent experiments with several replicates each. Statistical analysis was done using *t*-test. Scale bar (f) is 10 *μ*m; n.s. = not significant.

The next question was to investigate intracellular signaling events upon plasma-induced H2AX phosphorylation. Many pathways leading to *γ*H2AX have been unraveled [36], and we used several inhibitors in preliminary tests (Supplementary [Supplementary-material supplementary-material-1]–[Supplementary-material supplementary-material-1]). One promising candidate was SB202190, a p38-MAPK inhibitor, which gave a significant decrease in *γ*H2AX induction for all ROS but not for UV treatment (Figure 3(a)). Similar results were achieved with Z-VAD-FMK, a pan-caspase inhibitor (Figure 3(b)), but only a no-significant reduction was observed with KU59933 for plasma conditions (Supplementary [Supplementary-material supplementary-material-1]), an ataxia telangiectasia mutated (ATM) inhibitor. This suggests that plasma-induced *γ*H2AX induction is a result of apoptosis induction rather than of plasma-derived ROS directly traveling through the cells and eventually to the nucleus to confer DNA damage. To confirm functionality of Z-VAD-FMK on inhibiting apoptosis, we measured caspase 3 and 7 activity (Figure 2(c)). Quantification at 4 h (Figure 2(d)) and 24 h (Figure 2(e)) revealed a significant increase in nonapoptotic cells with all treatment modalities. The fact that UV-induced apoptosis but not H2AX phosphorylation was abrogated with Z-VAD-FMK suggests that *γ*H2AX induction was regulated by pathways not related to apoptosis, which was not the case for ROS conditions. Finally, using confocal laser scanning microscopy, we confirmed that *γ*H2AX foci were majorly present in apoptotic cells showing fragmented nuclei (Figure 3(f)). Taken together, plasma and ROS but not UV-induced *γ*H2AX was largely dependent on stress (p38-MAPK) and apoptosis (caspase) signaling pathways.

### 3.4. Plasma-Mediated *γ*H2AX Induction Does Not Correlate to DNA DSB-Related Micronuclei

**Figure 4:**
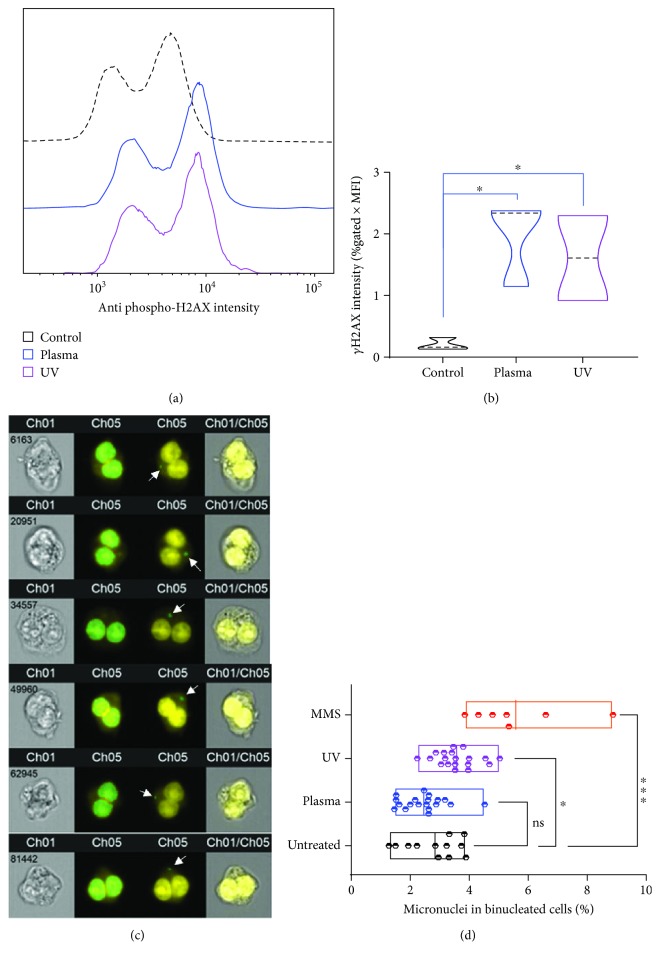
Correlation of *γ*H2AX expression and micronuclei formation. (a, b) Representative histogram overlay *γ*H2AX fluorescence (a) and its quantification (b) in control as well as plasma and UV-treated cells. (c) Representative images (left: brightfield, right: overlay) of high-throughput imaging cytometry of TK6 cells to analyze draq5-stained nuclei (yellow) with masks for binucleated cells (middle left large green overlays) and micronuclei (middle right small green overlays with white arrows). (d) Quantification of micronuclei in binucleated TK6 cells that were left untreated or exposed to plasma, UV light, or methyl methanesulfonate (MMS). Data are from two (b) and three to four (d) independent experiments with several replicates each. Data show violin plots (b) and min-to-max floating bars (d). Statistical analysis was done with one-way ANOVA.

To confirm that plasma and ROS-induced *γ*H2AX foci were a consequence of ROS-induced stress signaling and apoptosis-induced DNA DSBs rather than markers of direct ROS-induced DNA DSBs, we performed a functional assay on DNA DSBs, the cytokinesis-block micronucleus (MN) assay. Sufficient numbers of DNA DSBs lead to replication errors during DNA synthesis in G2 phase cells, which converts into micronuclei formation as genotoxic endpoint measurement [46]. The addition of cytochalasin B blocks cytoplasmic division due to inhibition of network formation of actin filaments. The result is an enrichment of binucleated (G2 phase) cells (BNCs) that can be quantitatively assessed with different DNA stains (Supplementary [Supplementary-material supplementary-material-1]). Imaging (Supplementary [Supplementary-material supplementary-material-1]) and quantification of BNCs (Supplementary [Supplementary-material supplementary-material-1]) confirmed this principle, and we decided to use draq5 for subsequent experiments. Another observation was that the plasma and UV treatment time utilized in experiments were too toxic in combination with cytochalasin B, leaving only few cells to analyze at 48 h posttreatment. Therefore, plasma and UV treatment time was reduced to 2.5 s and 24 s, respectively, which still generated significantly more *γ*H2AX signal compared to untreated control (Figures 4(a) and 4(b)). By applying customized mathematical operands, several masks were develop to clearly identify and quantify BNCs as well as MN within the population of BNCs in an algorithm-based, unbiased fashion (Figure 4(c)) across millions of cells. Final analysis showed a significant increase in MN formation with UV but not plasma treatment. A chemical genotoxic agent (MMS) was installed as additional control in this experiment, which differed significantly from untreated cells (Figure 4(d)). As additional quality control, the average number of cells analyzed in these experiments was quantified and was similar among all conditions (Supplementary [Supplementary-material supplementary-material-1]). In sum, MN formation correlated with *γ*H2AX induction for UV treatment, which directly acts on cellular DNA, but not for plasma treatment, which acts on cells by generating exogenous ROS that subsequently diffuse to cells to exert their stress and apoptosis-inducing but not directly DNA-damaging function.

## 4. Discussion

DNA damage and the DNA damage response are important elements in medical treatment modalities, such as radiation therapy and chemotherapeutic drugs in several medical fields including in oncology [3]. For example, the anticancer drug doxorubicin can both induce DNA DSBs and generate ROS, leading to *γ*H2AX induction [47–49]. Cell metabolism, oxidative stress, and DNA damage are often intertwined and difficult to study independently, leading to the general assumption of *γ*H2AX foci being a hallmark of DNA DSBs and damage. We here provide evidence that exogenous ROS added experimentally or generated with cold physical plasmas led to *γ*H2AX induction only in case of apoptosis, and without long-term genotoxic effects. In such setting, the presence of *γ*H2AX may be a consequence of low oxidative stress rather than an indicator of DNA damage. Recent data suggest *γ*H2AX to even play in pivotal role in antioxidant defense signaling [50]. H2AX-knockout cells showed increase endogenous ROS levels and failed to activate the antioxidant response elements through nuclear factor E2-related factor 2, Nrf2 [51], along with mitochondrial damage [29].

In our study, we used UV-B radiation as positive control for *γ*H2AX and micronuclei induction. In contrast to exogenous ROS (plasma, H_2_O_2_, or HOCl), occurrence of *γ*H2AX was independent of the use of antioxidants and maximum in S-phase cells. The latter corroborates previous findings, where also a repression of *γ*H2AX induction with PI3K inhibition using 5 mM of caffeine was found [52]. We did not find such decrease with PI3K inhibitors wortmannin and Ly294002, which may be due to different cell types and concentrations tested. Another study found ATR kinase to be the crucial determinant for UV-induced H2AX phosphorylation and confirmed our findings of maximum *γ*H2AX induction at 2 h after treatment prior to onset of intermediate stages of apoptosis where *γ*H2AX dramatically increases [53]. The ability of UV-B enhancing the frequency of MN in cells has been reported before [54, 55]. Interestingly, catalase decreased oxidation in UV-treated cells but not H2AX phosphorylation. UV generates ROS in the intracellular as well as extracellular compartment [56]. As the experimentally added catalase only acts in the extracellular compartment, a partial protection from oxidation was observed without protecting the DNA (intracellular compartment) from UV-mediated ROS and damage.

With exogenous ROS (plasma, H_2_O_2_, HOCl), we observed a strong dependence on p38-MAPK signaling and caspase activation in TK6 cells. This is in line with previous findings using oxaliplatin, an antitumor tumor drug leading to DNA DSBs, where pretreatment of cells with SB202190 (p38-MAPK inhibitor) and Z-VAD-FMK (caspase inhibitor) abrogated oxaliplatin-induced *γ*H2AX induction and apoptosis in HCT116 cells [57]. In leukemia cells, it was reported that *γ*H2AX (or blockage of H2AX phosphorylation by SB202190) expression sensitizes cells to apoptosis, suggesting a pivotal role of *γ*H2AX in cell death signaling [58]. This is supported by findings with H2AX-knockout fibroblasts, which upon UV treatment activates caspase 3 but cannot activate caspase-activated DNAse (CAD), a crucial step in DNA fragmentation required for apoptosis [59]. Another form of regulated cell death (RCD) leading to widespread DNA fragmentation is parthanatos, but this mode of RCD is independent of apoptotic caspases [60]. Hence, parthanatos is not a main mechanism in our study because we observed apoptosis-induced and caspase-dependent DNA fragmentation. Hence, *γ*H2AX is heavily intertwined in cell death signaling and our study supports this notion as caspase inhibition abrogated both H2AX phosphorylation and apoptosis.

Heavy H2AX phosphorylation indicates toxic numbers of DNA DSBs in, e.g., ionizing radiation, UV treatment, replication, and apoptosis. Contrasting radiation-induced DNA DSBs, we found *γ*H2AX to be a consequence of ROS-induced apoptosis rather than its cause. T lymphocytes are very sensitive towards (plasma-induced) oxidative stress [61–64]. This is due to (low-dose) ROS acting as proapoptotic and redox-signaling agents and not as toxic molecules *per se* [65]. In our study, e.g., few micromolar H_2_O_2_ on 250,000 cells were sufficient to induce cell death. The concentrations of, e.g., H_2_O_2_ used in many genotoxicity studies are 10-50-fold higher at lower absolute cell numbers [66–68]. So far, only few studies in plasma medicine investigated genotoxicity in cells and tissues using non-*γ*H2AX assays, and the once that have did not report mutagenic effects of plasma treatment. Using the hypoxanthine-guanine phosphoribosyl-transferase (HPRT) assay and the MN assay in V79 cells, plasma treatment failed to induce mutagenic effects when exposing cells to the plasma of the kINPen or its products [69], or when using another plasma source designed for medical application at microbicidal concentrations [70, 71]. Modulation of the feed gas composition of the kINPen yielded similar results [43]. Micronuclei have also been quantified *in vivo* in the hen's egg model after treatment with cyclophosphamide or methotrexate (as positive control), or cold physical plasma generated with the kINPen. Results showed the absence of genotoxicity-indicating MN with plasma treatment compared to positive controls [72]. In human tissue exposed to cold physical plasma at short, intermediate, and long treatment times, an increase of *γ*H2AX was not observed when compared to untreated control tissue [73]. Moreover, in a one-year follow-up of mice (human equivalent of 65 years) treated six times with plasma within 2 weeks, no occurrence of malignant lesions was observed anywhere in the body as shown by immunohistochemistry and PET-CT and MRI scan [74].

ROS can also induce lipid peroxidation [75], which can contribute to DNA damage. However, for H_2_O_2_—which gave large *γ*H2AX signals—concentration reported for lipid peroxidation exceeds the one used in our study by 50-fold [76]. For lipid vesicles, H_2_O_2_ is ineffective in the absence of the Fenton reaction [77], even at concentration 5000 times of that used in our work [78]. For H_2_O_2_ treatment in our study, caspase inhibition decreased H_2_O_2_-incuded *γ*H2AX to background levels, which argues against H_2_O_2_-induced lipid peroxidation as major mechanism for DNA DSBs. Moreover, if lipid peroxidation would have contributed to plasma-induced DNA damage, e.g., via UV-mediated photolysis and hydroxyl radical generation, addition of catalase would not have abrogated the increase observed in *γ*H2AX.

Our results are of relevance for medical plasmas, while findings may be different for industrial plasma applications. Atmospheric and room temperature plasma (ARTP) has been recently described as tool for mutation breeding in microorganisms [79–81]. Its efficacy seemed greater than that of several conventional methods, e.g., chemicals and UV radiation [82], and ARTP can also be combined with such methods [83]. As result, ARTP was found to increase production of, for instance, *α*-ketoglutaric acid [84], biofuel [85], polysaccharides [86, 87], arachidonic acid [88], erythritol [89], l-arginine [90], alkaline *α*-amylase [91, 92], d-lactic acid [93], lycopene [94], and carotenoids and lipids [95] in different types of microorganisms. However, it is important to mention that these plasma sources are not intended for medical applications. Hence, they may differ substantially in their geometry and power consumption, leading to enhanced UV radiation, ROS generation, and electrical discharges. In our study, we used a low-energy [96] and clinically effective [8, 97, 98] plasma jet complying to European regulations (e.g., generation of ozone, UV, and leak currents), which is not true for the majority of other plasma devices reported in experimental studies.

A limitation of our study is the lack of short-term kinetic measurements of *γ*H2AX and (onset of) apoptosis. Moreover, *γ*H2AX foci expand over time [99], making it difficult to distinguish between many foci with low intensity vs. few foci with high intensity (and anything in between) in our flow cytometry data. Additionally, other proteins including Nbs1, 53BP1, and Brca1 are recruited to and hence mark DNA DSBs [31], which may be investigated in future studies.

## 5. Conclusion

Cold physical plasma-induced *γ*H2AX marks DNA DSBs as a consequence of oxidative stress and apoptosis *in vitro*. Upon blocking apoptosis and p38 MAPK signaling, increased *γ*H2AX with plasma treatment was abolished, arguing that H2AX phosphorylation is a secondary event in redox or apoptotic signaling rather than a primary consequence of direct ROS-mediated DNA damage. In contrast to UV treatment, exposure to plasma did not correlate with long-lasting genotoxic effects as indicated using the micronucleus assay. Hence, *γ*H2AX measurements in plasma medical research should be interpreted with care, keeping in mind the pleiotropic roles of this molecule in redox sensing and apoptotic pathways.

## Data Availability

The data used to support the findings of this study are available from the corresponding author upon request.

## Supplementary Materials

Supplementary Materials Supplementary Figure S1: (a) metabolic activity in TK6 cells at 24 h, 48 h, and 72 h after exposure to *γ*H2AX-inducing agents; (b) quantification of nonterminally dead (DAPI-) TK6 cells at 6 h after exposure to different concentrations or treatment times of *γ*H2AX-inducing agents; (c) dilution series of anti-*γ*H2AX antibody in control and UV-treated TK6 cells to obtain optimal antibody concentration; (d) kinetic experiment of *γ*H2AX intensity in control or plasma and UV-treated TK6 cells; (e) *γ*H2AX intensity in each cell cycle phase of untreated cells incubated with vehicle, catalase, and GSH; (f) incubation with antioxidant NAC increased *γ*H2AX intensity with all treatments as well as in untreated cells and therefore was not used in this study. Supplementary Figure S2: serial dilutions of inhibitors. (a) SB202190 (p38-MAPK inhibitor) was used in the study (Figure 3(a)); (b) SP600125 (JNK inhibitor) disqualified due to the increase of *γ*H2AX in untreated cells upon incubation with the drug; (c) Ly294002 (PI3K inhibitor) did not decrease *γ*H2AX in plasma-treated cells; (d) wortmannin (PI3K inhibitor) disqualified due to the increase of *γ*H2AX in untreated cells upon incubation with the drug; (e) Z-VAD-FMK (pan-caspase inhibitor) was used in the study (Figure 3(b)); (f) KU55933 (ATM-kinase inhibitor) showed a small decrease in *γ*H2AX, which (g) was not significant with any treatment in three independent repeats. Supplementary Figure S3: optimization of the micronucleus assay. (a) cell cycle analysis of untreated and treated (cytochalasin B for 24 h) TK6 cells with different DNA-binding dyes; (b) representative images of cells in brightfield (Ch05 or Ch01) and DNA-binding dye (Ch1 or Ch5) showing binucleated cells (BNCs); (c) quantification of binucleated cells stained with four different DNA-binding dyes via a complex software algorithm designed to create a specific set of masks to BNCs as described before [1], we aimed at a low amount of BNCs to obtain a high specificity for micronuclei leading to the choice of draq5 for main experiments; (d) mean total count of cells in samples from each condition.
