# Supplementary material for: Elevated H2AX Phosphorylation Observed with kINPen Plasma Treatment Is Not Caused by ROS-Mediated DNA Damage but Is the Consequence of Apoptosis
Source: Oxid Med Cell Longev. 2019 Sep 19;2019:8535163. doi: 10.1155/2019/8535163 (PMC6770374; doi:10.1155/2019/8535163)
Supplement: Supplementary Materials — Supplementary Figure S1: (a) metabolic activity in TK6 cells at 24 h, 48 h, and 72 h after exposure to γH2AX-inducing agents; (b) quantification of nonterminally dead (DAPI-) TK6 cells at 6 h after exposure to different concentrations or treatment times of γH2AX-inducing agents; (c) dilution series of anti-γH2AX antibody in control and UV-treated TK6 cells to obtain optimal antibody concentration; (d) kinetic experiment of γH2AX intensity in control or plasma and UV-treated TK6 cells; (e) γH2AX intensity in each cell cycle phase of untreated cells incubated with vehicle, catalase, and GSH; (f) incubation with antioxidant NAC increased γH2AX intensity with all treatments as well as in untreated cells and therefore was not used in this study. Supplementary Figure S2: serial dilutions of inhibitors. (a) SB202190 (p38-MAPK inhibitor) was used in the study (Figure 3(a)); (b) SP600125 (JNK inhibitor) disqualified due to the increase of γH2AX in untreated cells upon incubation with the drug; (c) Ly294002 (PI3K inhibitor) did not decrease γH2AX in plasma-treated cells; (d) wortmannin (PI3K inhibitor) disqualified due to the increase of γH2AX in untreated cells upon incubation with the drug; (e) Z-VAD-FMK (pan-caspase inhibitor) was used in the study (Figure 3(b)); (f) KU55933 (ATM-kinase inhibitor) showed a small decrease in γH2AX, which (g) was not significant with any treatment in three independent repeats. Supplementary Figure S3: optimization of the micronucleus assay. (a) cell cycle analysis of untreated and treated (cytochalasin B for 24 h) TK6 cells with different DNA-binding dyes; (b) representative images of cells in brightfield (Ch05 or Ch01) and DNA-binding dye (Ch1 or Ch5) showing binucleated cells (BNCs); (c) quantification of binucleated cells stained with four different DNA-binding dyes via a complex software algorithm designed to create a specific set of masks to BNCs as described before [1], we aimed at a low amount of BNCs to obtain a high specificity for [file 8535163.f1.docx]

# Cold physical plasma-induced H2AX phosphorylation relates to oxidative stress sensing and apoptosis rather than DNA damage

Sander Bekeschus^1,*^, Clarissa S. Schütz^1,2^, Felix Nießner^1^, Kristian Wende^1^, Klaus‑Dieter Weltmann^1^, Nadine Gelbrich^2^, Thomas von Woedtke^1,3^, Anke Schmidt^1^, Matthias B. Stope^2^

1. ZIK *plasmatis*, Leibniz Institute for Plasma Science and Technology (INP Greifswald), Greifswald, Germany
2. Department of Urology, Greifswald University Medical Center, Greifswald, Germany
3. Institute for Hygiene and Environmental Medicine, Greifswald University Medical Center, Greifswald, Germany

*correspondence: [sander.bekeschus@inp-greifswald.de](mailto:sander.bekeschus@inp-greifswald.de)

Supplemental information:

The supplemental information provide additional kinetic data and controls on metabolic activity and H2AX phosphorylation (Figure S1), serial dilutions on inhibitors (Figure S2), and assay setup and validation data for the micronucleus test (Figure S3) in TK6 cells in response to the different ROS and UV treatment.


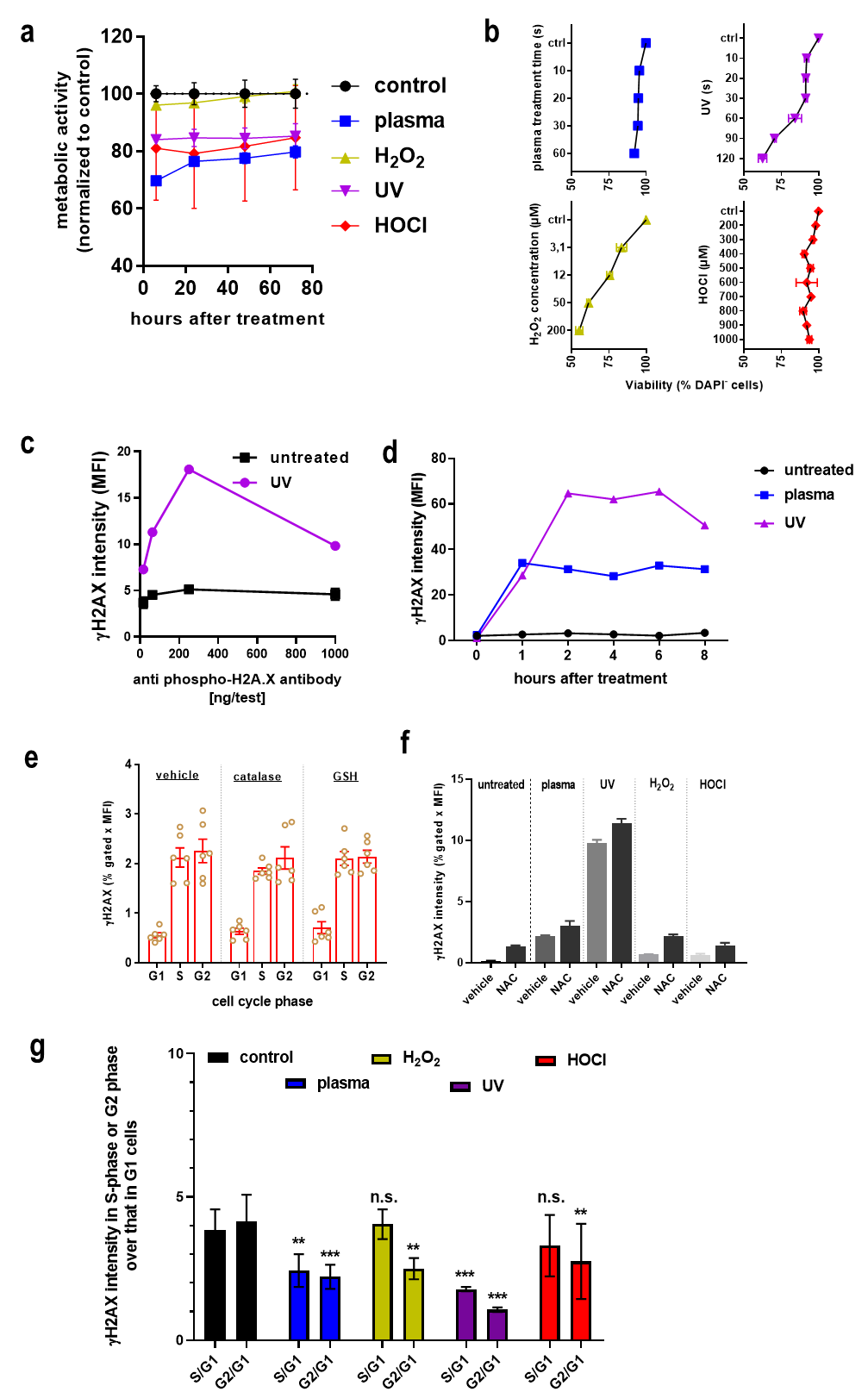


Supplementary figure S1: (a) metabolic activity in TK6 cells at 24h, 48h, and 72h after exposure to γH2AX-inducing agents; (b) quantification of non-terminally dead (DAPI^-^) TK6 cells at 6h after exposure to different concentrations or treatment times of γH2AX-inducing agents; (c) dilution series of anti-γH2AX antibody in control and UV-treated TK6 cells to obtain optimal antibody concentration; (d) kinetic experiment of γH2AX intensity in control or plasma and UV-treated TK6 cells; (e) γH2AX intensity in each cell cycle phase of untreated cells incubated with vehicle, catalase, and GSH; (f) incubation with antioxidant NAC (adjusted to physiological pH) increased γH2AX intensity with all treatments as well as in untreated cells and therefore was not used in this study; (g) ratio of γH2AX intensity in S or G2 cells over that of G1 cells for each treatment or control cells with two-way anova with Sidak-post-hoc-test for statistical comparison of treatments (plasma, H_2_O_2_, UV, HOCl) compared to control. ** = p<0.01, *** = p<0.001.


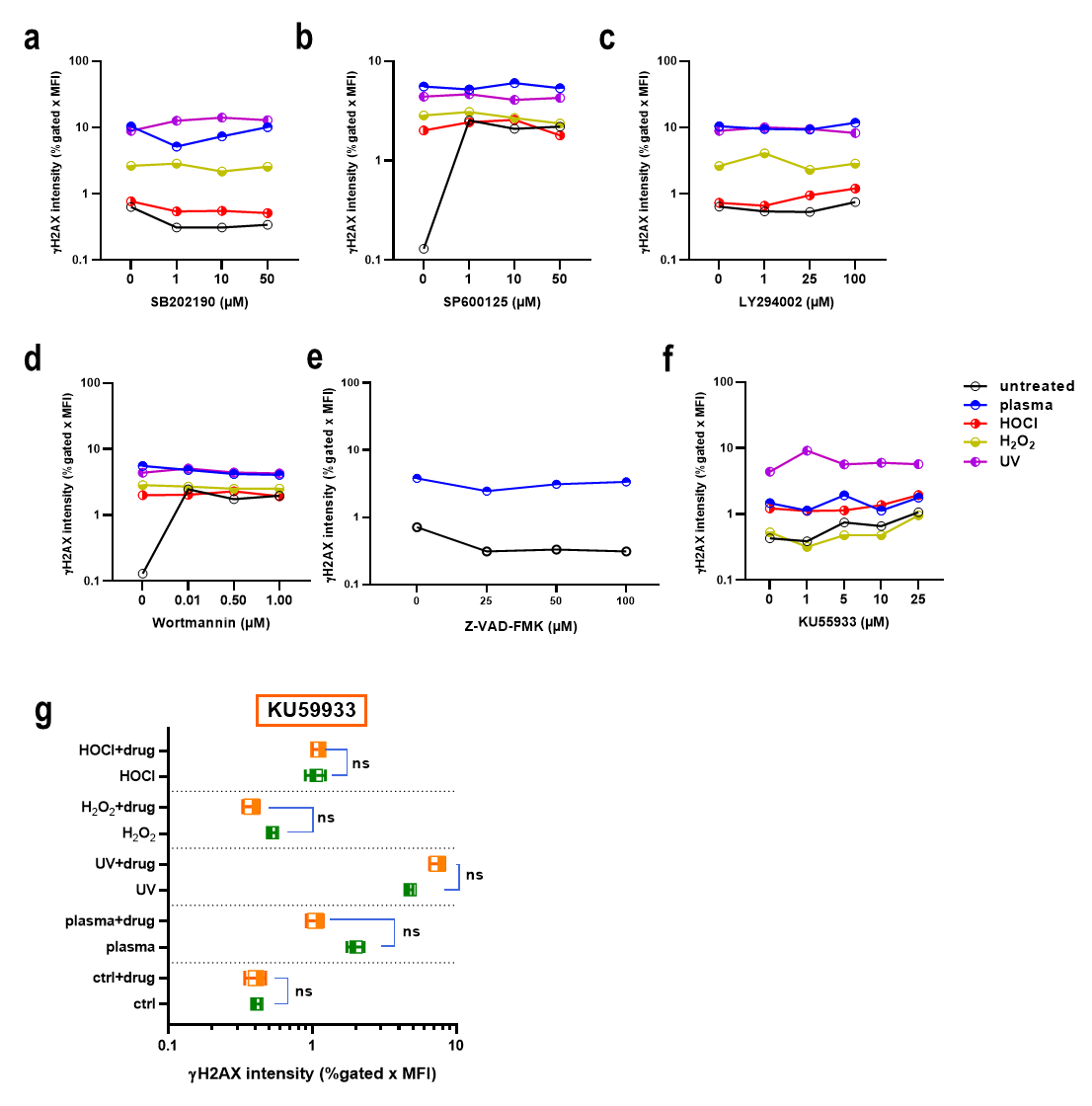


Supplementary figure S2: Serial dilutions of inhibitors. (a) SB202190 (p38-MAPK inhibitor) was used in the study (figure 3a); (b) SP600125 (JNK inhibitor) disqualified due to the increase of γH2AX in untreated cells upon incubation with the drug; (c) Ly294002 (PI3K inhibitor) did not decrease γH2AX in plasma treated cells; (d) wortmannin (PI3K inhibitor) disqualified due to the increase of γH2AX in untreated cells upon incubation with the drug; (e) Z-VAD-FMK (pan caspase inhibitor) was used in the study (figure 3b); (f) KU55933 (ATM-kinase inhibitor) showed a small decrease in γH2AX, which (g) was not significant with any treatment in three independent repeats.


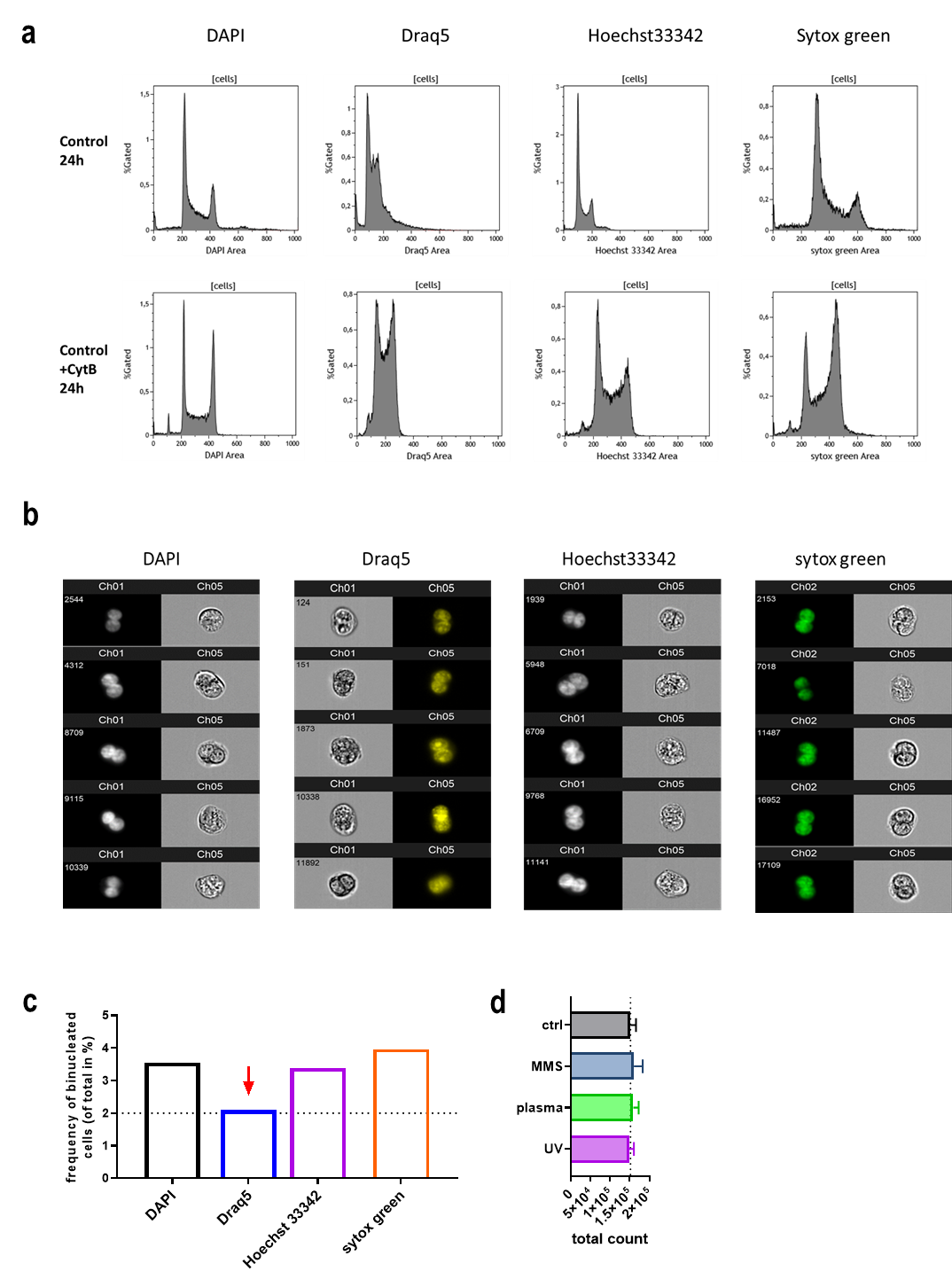


Supplementary figure S3: Optimization of the micronucleus assay. (a) cell cycle analysis of untreated and treated (cytochalasin B for 24h) TK6 cells with different DNA-binding dyes; (b) representative images of cells in brightfield (Ch05 or Ch01) and DNA-binding dye (Ch1 or Ch5) showing binucleated cells (BNCs); (c) quantification of binucleated cells stained with four different DNA-binding dyes via a complex software algorithm designed to create a specific set of masks to BNCs as described before (1), we aimed at a low amount of BNCs to obtain a high specificity for micronuclei leading to the choice of draq5 for main experiments; (d) mean total count of cells in samples from each condition.
